# The miR-17-5p microRNA is a key regulator of the G1/S phase cell cycle transition

**DOI:** 10.1186/gb-2008-9-8-r127

**Published:** 2008-08-14

**Authors:** Nicole Cloonan, Mellissa K Brown, Anita L Steptoe, Shivangi Wani, Wei Ling Chan, Alistair RR Forrest, Gabriel Kolle, Brian Gabrielli, Sean M Grimmond

**Affiliations:** 1 Institute for Molecular Bioscience, The University of Queensland, Carmody Road, St Lucia, 4072, Australia; 2 Diamantina Institute for Cancer, Immunology and Metabolic Medicine, Princess Alexandra Hospital, Ipswich Road, Woolloongabba, 4102, Australia; 3 Genomic Sciences Center, RIKEN Yokohama Institute, Yokohama, 230-0045 Japan

## Abstract

**Background:**

MicroRNAs are modifiers of gene expression, acting to reduce translation through either translational repression or mRNA cleavage. Recently, it has been shown that some microRNAs can act to promote or suppress cell transformation, with miR-17-92 described as the first oncogenic microRNA. The association of miR-17-92 encoded microRNAs with a surprisingly broad range of cancers not only underlines the clinical significance of this locus, but also suggests that miR-17-92 may regulate fundamental biological processes, and for these reasons miR-17-92 has been considered as a therapeutic target.

**Results:**

In this study, we show that miR-17-92 is a cell cycle regulated locus, and ectopic expression of a single microRNA (miR-17-5p) is sufficient to drive a proliferative signal in HEK293T cells. For the first time, we reveal the mechanism behind this response - miR-17-5p acts specifically at the G1/S-phase cell cycle boundary, by targeting more than 20 genes involved in the transition between these phases. While both pro- and anti-proliferative genes are targeted by miR-17-5p, pro-proliferative mRNAs are specifically up-regulated by secondary and/or tertiary effects in HEK293T cells.

**Conclusion:**

The miR-17-5p microRNA is able to act as both an oncogene and a tumor suppressor in different cellular contexts; our model of competing positive and negative signals can explain both of these activities. The coordinated suppression of proliferation-inhibitors allows miR-17-5p to efficiently de-couple negative regulators of the MAPK (mitogen activated protein kinase) signaling cascade, promoting growth in HEK293T cells. Additionally, we have demonstrated the utility of a systems biology approach as a unique and rapid approach to uncover microRNA function.

## Abstract 2

Novel targets of the oncogenic miR-17-92 cluster have been identified and the mechanism of regulation of proliferation at the G1/S phase cell cycle transition via the miR-17-5p microRNA has been elucidated.

## Background

MicroRNAs (miRNAs) are short, non-coding, RNA regulators of gene expression that have been identified in a broad range of eukaryotes. In addition to regulating growth, development, differentiation, and metabolism in model organisms, some miRNAs have also been classified as tumor suppressors or oncogenes (reviewed in [1]).

The first reported and most well studied oncomiR is the human miR-17-92 polycistron: a cluster of seven miRNAs derived from the c-myc regulated c13orf25 locus at chromosome 13q31.3 [2]. miRNA 17-5p is homologous with two other miRNAs within this cluster (miRs 18 and 20), while miR-19a differs by only one nucleotide from miR-19b-1 [3]. The status of miR-17-3p as a functional miRNA is still controversial [4-6]. The entire cluster also has paralogues within the genome, at chromosome Xq26.2 (hsa-mir-106a, has-mir-18b, has-mir-20b, hsa-mir-19b-2, hsa-mir-92-2) and chromosome 7q22.1 (hsa-mir-106b, hsa-mir-93, hsa-mir-25) [2,3,5]. The former has been implicated in the progression of T-cell leukemia [7], while the latter has yet to be implicated in any disease state.

By contrast, over-expression of the mir-17-92 locus has been identified in lung cancers [3], chronic myeloid leukemias [6], B-cell and mantle cell lymphomas [2,8], hepatocellular tumors [9], bladder cancers [10], and breast, colon, pancreas, prostate, and stomach solid tumors [11]. Additionally, the mir-17-92 cluster appears to act as a tumor suppressor in some breast and ovarian cancer cell lines [12]. The association of miR-17-92 with a broad range of cancers not only underlines the clinical significance of this locus, but also suggests that miR-17-92 may regulate fundamental biological processes.

Although miRNAs are generally predicted to target hundreds of genes [13,14], experimental evidence of miRNA-mRNA interactions from the miR-17-92 cluster has been limited to a few key components. Previous work has confirmed that CDKN1B is regulated by the miR-17-92 cluster [15]; E2F1-3, NCOA3, and RBL2 are targets of hsa-mir-17-5p [5,12,16,17]; PCAF, RUNX1, and TGFBR2 are targets of both miR-17-5p and miR-20a [11,18-20]; CTGF is a target of miR-18a [21]; and PTEN and THBS1 are targets of miR-19a [21,22]. Many of these targets are known cell cycle regulators, although none of these interactions are sufficient to explain the oncogenic potential of this locus. The specific mechanisms of either the tumor suppressor or oncogenic activities of the miR-17-92 miRNAs remain unknown.

In this study, we employ a systems biology approach to uncover a large network of interacting genes that are directly targeted by miR-17-5p. We show that ectopic expression of miR-17-5p leads to dysregulation of normal cell cycle progression and a pro-proliferative response in HEK293T cells. For the first time, we show how this miRNA can drive both pro- and anti-proliferative signals, allowing for the switch between oncogenic and tumor suppressor activities.

## Results

### The mir-17-92 locus is cell cycle regulated

**Figure 1:**
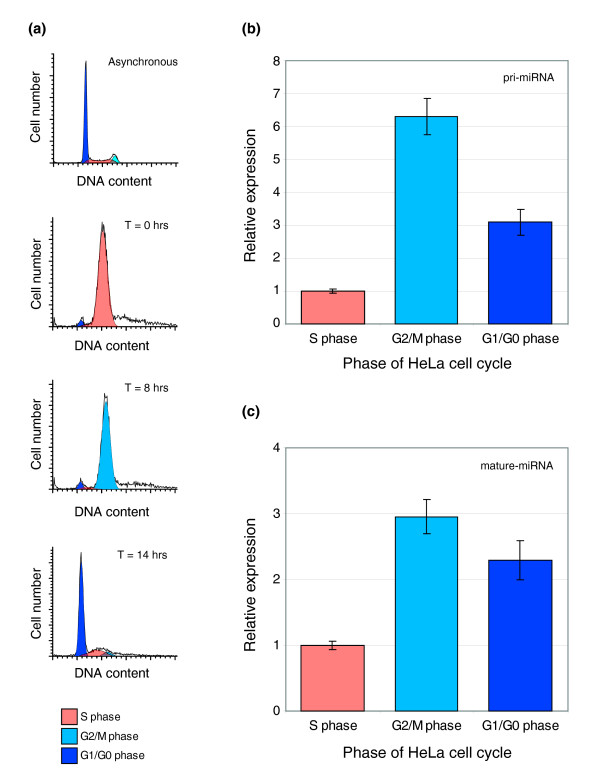
Phase enriched expression of the miR-17-92 locus. **(a) **HeLa cells were synchronized by double-thymidine block and synchrony was assessed by flow cytometry analysis of propidium iodide stained cells. DNA profiles are show from top to bottom as follows: asynchronous cells, S-phase cells (T = 0 h; synchrony >97%), G2/M phase cells (T = 8 h; synchrony >93%), and G1/GO phase cells (T = 14 h; synchrony >74%). Within each profile, cells classified as G1/G0 are depicted in dark blue, S-phase are depicted in orange, and G2/M are depicted in light blue. **(b) **Graph showing relative expression of miR17-92 pri-miRNAs in synchronized HeLa S phase, G2/M phase, and G1/G0 phase cell populations as assessed by qRT-PCR. **(c) **Graph showing relative expression of miR17-5p mature miRNAs in synchronized HeLa S phase, G2/M phase, and G1/G0 phase cell populations as assessed by qRT-PCR (mean ± SEM).

While previous studies have shown that the miR-17-92 locus is regulated by Myc and the E2F family of transcription factors, the regulation of this gene during the cell cycle has not yet been explored. To determine whether this locus was expressed in a phase-specific manner, we performed quantitative real-time PCR (qRT-PCR) on RNA isolated from synchronized G1/G0, S, and G2/M populations of HeLa cells (which express moderate amounts of miR-17-5p) to detect the expression of endogenous mir-17-92 pri-RNA. The synchrony of the cells was confirmed by flow cytometry analyses of their DNA profiles (Figure 1a). We found that the mir-17-92 locus is differentially expressed during the different stages of the HeLa cell cycle, and has its highest expression in G2/M (Figure 1b). Similarly, we find that the mature miR-17-5p miRNA follows the same profile as the pri-mRNA, with its highest expression in G2/M (Figure 1c). While it is clear that the miR-17-92 locus is not essential for cell cycle progression (as many cell lines do not express this gene), the phase enriched expression of this locus suggests that its biological function is cell cycle related.

### miR-17-5p is sufficient to drive a proliferative signal in HEK293T cells

Although the entire miR-17-92 locus has been implicated in the progression of tumor development, several groups have previously reported differences in absolute expression of the individual miRNAs from this cluster [2,3,11,15], and non-coordinated dynamic expression of these same miRNAs [6,16,20]. Together, these data suggest that although these miRNAs are derived from the same transcript, they are differentially regulated into their mature (active) form. As differential regulation may allow for different functions, we wondered whether individual miRNAs could drive the proliferative response seen with miR-17-92 over-expression [3,16,19], or whether this phenotype was caused by synergistic action of the entire cluster. To address this, we undertook functional network analysis using Ingenuity Pathways Analysis (IPA). By using this web-based tool, findings presented in more than 200,000 peer-reviewed publications could be queried to determine the biological functions of genes predicted to be targets of the miR-17-92 polycistron.

For each miRNA in the miR-17-92 cluster, we reviewed its target genes, as previously predicted by PicTar (a miRNA-mRNA interaction predictor based on thermodynamic potential and evolutionarily conserved target sites [13]). We used IPA to screen for potentially enriched functional categories for these gene sets. To gauge the robustness of these annotations, we performed parallel analyses on similarly sized randomly selected gene sets. A functional category was deemed significantly enriched if its IPA score was more than four standard deviations above the mean score determined for the random gene lists.

**Figure 2:**
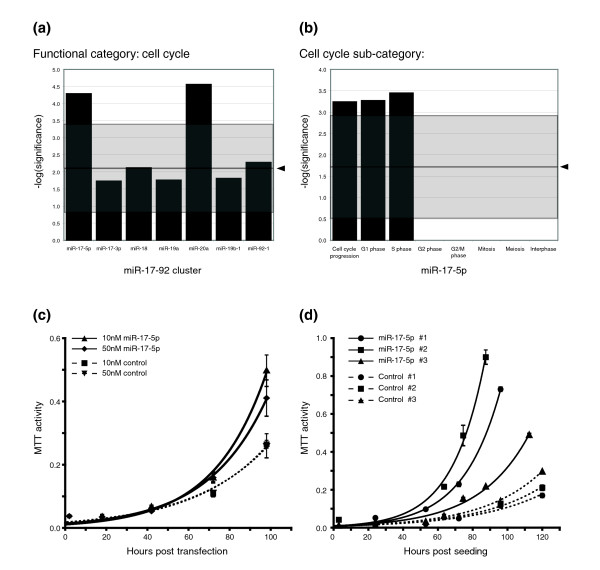
miR-17-5p is sufficient to drive a proliferative signal in HEK293T cells. **(a) **Graph displaying the significance of functional enrichment for PicTar predicted targets of miR-17-5p and miR-20a from the miR-17-92 cluster. Arrows indicate the mean significance of randomly selected gene sets of equivalent size, and the grey boxes show ± 4 standard deviations. **(b) **Graph displaying the significance of enrichment for genes acting at the G1/S cell cycle boundary. **(c) **Graph depicting the proliferation rates of HEK293T cells transiently transfected with miR-17-5p precursor dsRNA and those transfected with control dsRNA. **(d) **Graph depicting proliferation rates of HEK293T cells stably over-expressing plasmid-expressed miR-17-5p and HEK293T cells stably selected for the plasmid-control.

Of the seven miRNAs in the cluster, only miR-17-5p and miR-20a showed significant enrichment in any functional category, with both showing enrichment in genes that encode known cell cycle regulators, including four of the previously verified mir-17-5p targets; E2F1, NCOA3, PCAF, and RBL2 (Figure 2a). Within the cell cycle category, there was noted enrichment for genes associated with G1/S phase specific functions (Figure 2b): cell cycle progression, arrest in G1 phase, and entry into S phase. The enrichment of all these categories was more than five standard deviations away from what was seen for random sampling of similar sized sets of targets. The complete list of G1/S-phase related predictions is detailed in Additional data file 1. This analysis suggested that miR-17-5p may act by targeting genes involved in the G1 to S phase transition.

The miRNAs 17-5p and 20a share extensive sequence similarity, reflected in the significant overlap between predicted targets, however Hayashita *et al*. [3] found that miR-20a could not produce the hyper-proliferative phenotype in A549 cells. We therefore chose to focus our study further on the miR-17-5p-target network. We examined the effect of ectopic expression of miR-17-5p on HEK293T cell proliferation using a double-stranded RNA (dsRNA) miR-17-5p precursor, or a dsRNA negative control miRNA precursor. HEK293T cells have low levels of endogenous miR-17-5p expression, and miR-17-5p treated HEK293Ts proliferated faster post-transfection than the control cells (Figure 2c). To confirm this phenotype, we created vector based constructs with expression of miR-17-5p and created independent stable HEK293T cell lines with puromycin selection. The miR-17-5p activity of these cells was confirmed by luciferase reporter activity (Additional data file 2). The proliferative rate of the stable miR-17-5p cell lines (hereafter HEK293T-17-5p) was also significantly faster than the parental vector sequence alone (HEK293T-control; Figure 2d). Taken together, these results demonstrate that over-expression of miR-17-5p is sufficient to drive a proliferative signal in HEK293T cells.

### Over-expression of miR-17-5p alters the cell cycle profile of HEK293T cells

**Figure 3:**
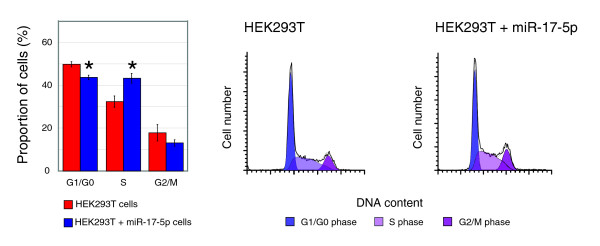
Over-expression of miR-17-5p alters the cell cycle profile of HEK293T cells. Graph and FACS plots displaying differences in cell cycle phases, as determined by FACS analysis, between normal and miR-7-5p expressing HEK293T cells. Cells over-expressing miR-17-5p have an altered cell cycle profile, with significantly less cells with G1/G0 DNA content, and significantly more with S-phase DNA content (mean ± SEM; asterisks indicate *p *≤ 0.05 in a Student's *t*-test).

We used flow cytometry to examine the DNA profiles of asynchronous populations of HEK293T-17-5p and control cell lines. HEK293T-17-5p cells differ significantly in their cell cycle distribution when compared to the control cell lines with a higher proportion of cells within S phase and less in G1/G0 (Figure 3). These data are consistent with an early exit from the G1/G0 stage, and can account for the rapid proliferation observed above. There was no observable difference in cell size between these cell lines or wild-type HEK293T cells as measured by flow cytometry forward scatter (data not shown). These data support the prediction above that miR-17-5p acts at the transition from G1 to S phase.

### Validation of predicted binding sites by luciferase assays

**Figure 4:**
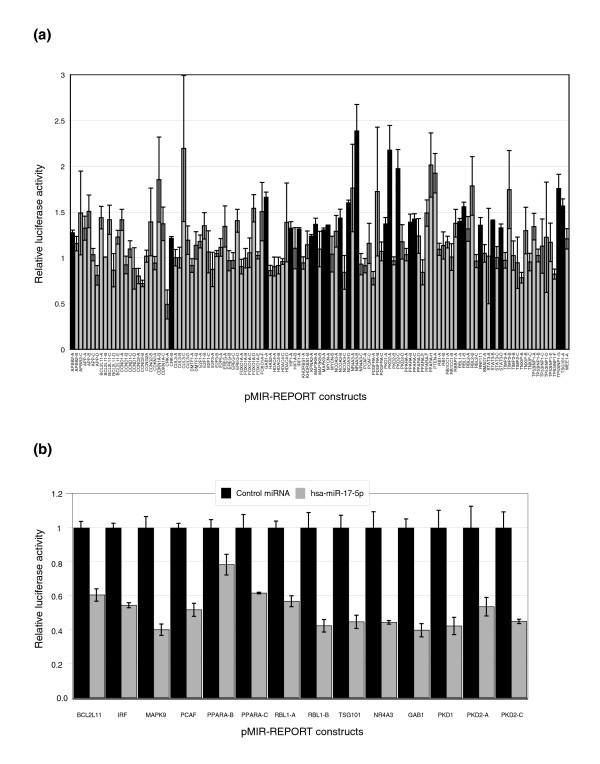
Validation of predicted binding sites by luciferase reporter assays. Synthetic oligonucleotides encoding 60 nucleotides that encompass predicted miRNA binding sites were cloned into luciferase reporter vectors. **(a) **These constructs were co-transfected into HEK293T-17-5p cells with a β-galactosidase expressing plasmid, and either a 17-5p 2'-O-Me ASO or a scrambled sequence ASO. Luciferase signals were normalized to β-galactosidase signals (as a control for transfection efficiency), and the mean and standard error relative to the scrambled ASO control are shown. Constructs that show a significant increase in luciferase expression with miR-17-5p ASO treatment (*p *≤ 0.05 in a Student's *t*-test) are indicated in black. **(b) **Selected constructs were co-transfected into HEK293T (wild-type cells that express very low levels of miR-17-5p) with a β-galactosidase expressing plasmid, and either a short dsRNA precursor for miR-17-5p or a negative control dsRNA precursor. Mean and standard errors of luciferase signals normalized to β-galactosidase activity are shown, and all sites except PPARA-B show significantly less luciferase activity with miR-17-5p treatment compared to control miRNA treatment (*p *≤ 0.05 in a Student's *t*-test).

In order to dissect the mechanisms of the miR-17-5p proliferative response, it was necessary to determine the endogenous mRNA targets of this miRNA. Predicted target sites were cloned into the 3' untranslated region (UTR) of a luciferase expressing vector, and transfected into the HEK293T 17-5p#1 stable cell line. Luciferase activity (directly proportional to translation from the plasmid) was measured in the presence of either a 2'-O-Methyl antisense oligoribonucleotide (ASO) specific for miR-17-5p, or a scrambled sequence ASO. If the luciferase expression of a test plasmid is inhibited by miRNA binding, then the presence of a specific miRNA ASO should result in an increase of luciferase activity. Figure 4a shows the assay results for 121 binding sites from 46 genes. We confirm interactions for NCOA3, and demonstrate an additional 18 targets of miR-17-5p, including GAB1, MAPK9, MYCN, PKD1, PKD2, RBL1, and TSG101, all of which are known to be involved in tumorigenesis and/or transformation of cells. As an additional test, we also transfected a number of these constructs into wild-type HEK293T cells with either a dsRNA miR-17-5p precursor, or a dsRNA negative control miRNA precursor. From 14 constructs tested, we find 13 with significantly less expression of luciferase when treated with the miR-17-5p dsRNA compared to the same constructs treated with the negative control dsRNA (Figure 4b). These data have confirmed our results above, and additionally confirmed BCL2L11 and PCAF as targets of miR-17-5p, all three of which are also known to be involved with cancer development.

**Figure 5:**
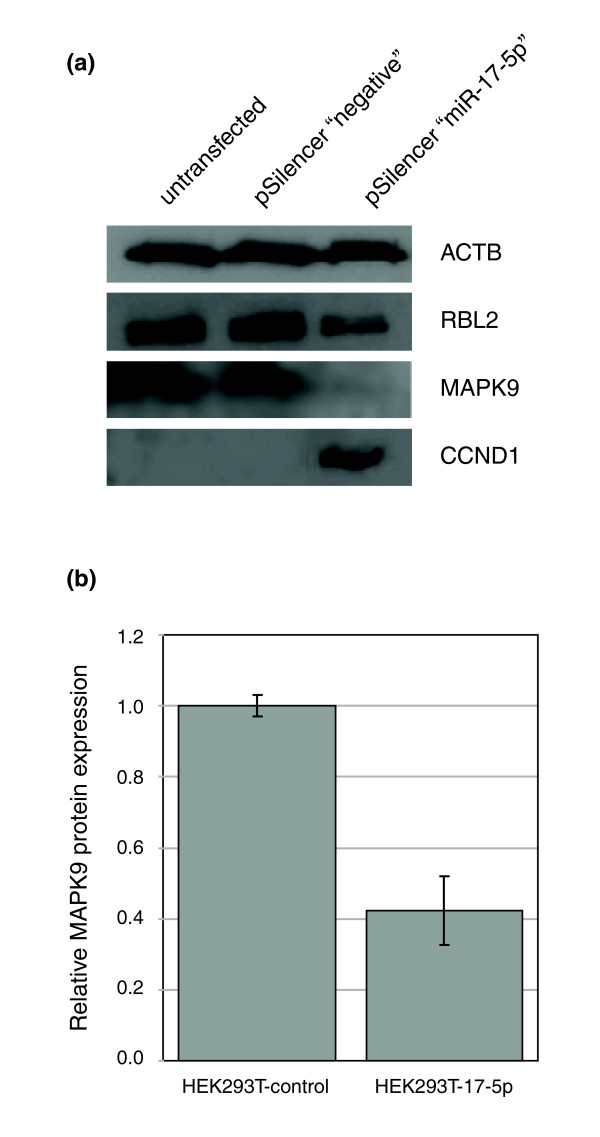
miR-17-5p targets MAPK9 translation. **(a) **Immunoblot analysis of miR-17-5p targets. Beta Actin (loading control), CCND1 and miR17-5p targets RBL2 and MAPK9 were assessed in untransfected, vector transfected and miR-17-5p transiently transfected lines. RBL2 and MAPK9 show lower protein levels while CCDN1 protein levels were dramatically increased in the miR17-5p expressing cell line. **(b) **Quantification of MAPK9 expression levels (assessed by immunoblot) in HEK293T-17-5p cell lines, and vector control cell lines grown under the same conditions. Mean and standard errors of independent experiments are shown (*p *= 0.02 in a Student's *t*-test).

Although we did not confirm the known interactions of E2F1 and RBL2 in these assays, we note that the single-site approach taken here will not detect a synergistic effect of multiple miRNA binding sites in the 3'UTR. Our assay shows that both E2F1 and RBL2 have multiple sites with miR-17-5p binding potential, although none reach significance individually. As we have confirmed the translational repression of both E2F1 and RBL2 in this system (data not shown, and Figure 5a, respectively), we applied this threshold to our results, and identified another four targets (APP, CDKN1A, EREG, and CUL3) that may also be regulated by miR-17-5p.

### MAPK9 translation is targeted by miR-17-5p

MAPK9 (more commonly known as JNK2) is an important member of the mitogen activated protein kinase (MAPK) family. MAPK9 is a negative regulator of cellular proliferation through a protein-protein interaction with its substrate JUN, targeting this transcription factor for protein-degradation. Knockout of MAPK9 stabilizes the JUN protein, resulting in increased CCND1 expression and rapid exit from G1 [23]. Our finding that miR-17-5p is capable of interacting with sequence in the 3'UTR of MAPK9 mRNA suggests that MAPK9 could be an important contributor to the hyper-proliferative phenotype caused by miR-17-5p. To examine this further we assessed the level of endogenous MAPK9 and CCND1 proteins after transient transfection with the miR-17-5p plasmid. We used protein expression of RBL2 as a positive control for miR-17-5p activity, and ACTB levels as a control for loading (Figure 5a). We see RBL2 and MAPK9 protein levels reduced in cells transfected with the miR-17-5p plasmid, but not with the plasmid control. MAPK9 protein levels are also significantly decreased in stable HEK293T-17-5p cell lines (Figure 5b). Additionally, we see an increase in CCND1 protein expression, confirming that de-coupling of the MAPK pathway from G1/S transition could contribute to our hyper-proliferative phenotype.

### miR-17-5p targets both suppressors and promoters of cellular proliferation

Amongst the confirmed targets of miR-17-5p are several inhibitors of cellular proliferation (such as TSG101, RBL1, and MAPK9), and their suppression is consistent with the pro-proliferative phenotype observed in HEK293T-17-5p cells. Conversely, several known promoters of cellular proliferation (such as MYCN, NCOA3, and NR4A3) were also found to be targets of miR17-5p, results that are not consistent with our pro-proliferative phenotype. In order to understand this apparent contradiction, we used IPA to examine known relationships between the targets of miR-17-5p (Additional data file 3) [24]. We find that this is a highly interacting network, comprising of many known transcriptional regulators that have known protein-DNA interactions with other members of the network.

In mammalian cells, miRNAs generally affect the protein output of a gene by inhibiting the translation of the mRNA. However, by changing the levels of a transcriptional regulator, a miRNA can indirectly affect the levels of mRNAs from other genes, which may include other targets of the miRNA. If the mRNA levels of a miRNA target are increased sufficiently, then this target will be able to overcome the effects of translational suppression, and maintain or increase protein levels. In the case of miR-17-5p, if proliferation-inhibitors suppress the mRNA levels of proliferation-promoters, then the consequential reduction of inhibitor-protein would lead to an increased level of promoter-mRNA, stabilizing the pro-proliferative signal. An example of this exists in our network - STAT3 protein (proliferation-inhibitor) inhibits the transcription of IRF1 mRNA (proliferation-promoter) [25].

**Figure 6:**
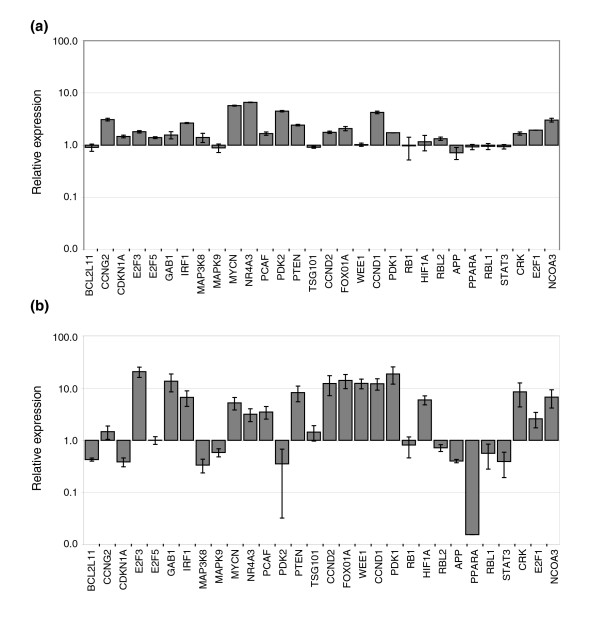
miR-17-5p perturbation of the transcriptional regulator network. qRT-PCR analysis of G1/S network mRNA levels, including 20 confirmed targets of miR-17-5p. **(a) **Cells transiently transfected with miR-17-5p dsRNA. **(b) **Cells with stable over-expression of plasmid-encoded miR-17-5p. In each case, the level of expression has been normalized to HPRT, and the means and standard errors are shown relative to the negative control.

We used qRT-PCR to examine the mRNA levels of 20 confirmed miR-17-5p targets, 3 possible miR-17-5p targets, and 7 other cell cycle related genes (CCND1, CCND2, CCNG2, E2F3, E2F5, RB1, and WEE1) in HEK293T cells with both transient (Figure 6a) and stable (Figure 6b) over-expression miR-17-5p. By examining both the transient and stable states, we are able to discriminate secondary effects of the miRNA (changes arising from miRNA suppression of transcriptional regulators) from tertiary effects (changes arising from secondary changes). For example, IRF1 mRNA was increased 2.6-fold in transiently transfected lines, likely to result as a secondary effect of STAT3 translational suppression (levels of STAT3 mRNA did not change in either transient or stable systems). Other changes in mRNA levels were clearly tertiary effects, such as E2F3 and WEE1 mRNA levels.

**Figure 7:**
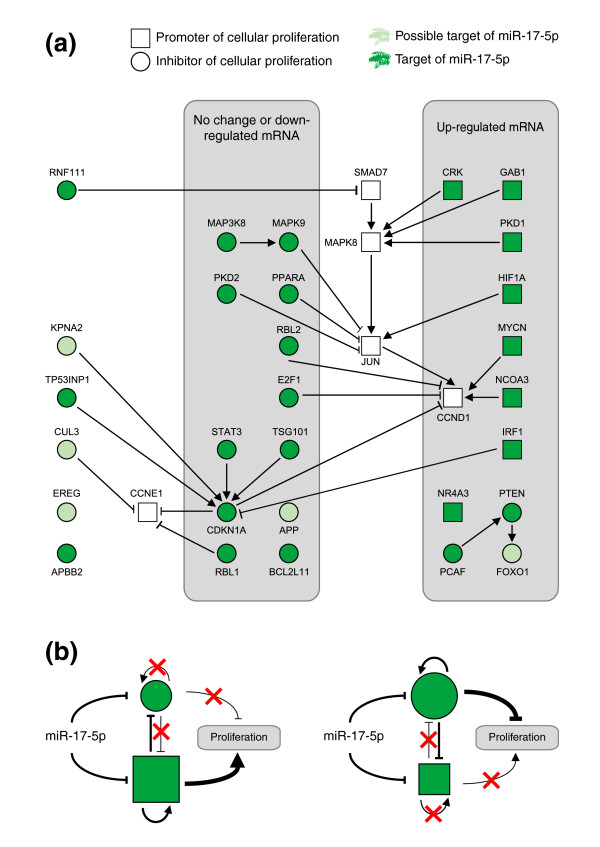
Network model summarizing the role of miR-17-5p in promoting cellular proliferation. **(a) **An integrated network model of results presented in this study. Each node present is either a possible (light green) or a confirmed/literature supported target of miR-17-5p (dark green). The shape of each node reflects whether the gene product encodes a pro-proliferative signal (square) or anti-proliferative signal (circle). The edges represent published interactions between nodes and are classified as either activation (arrowheads) or inhibition (perpendicular ends). All edges are supported by at least one reference from the literature. Finally, nodes whose mRNA levels have been examined by qRT-PCR appear in the grey boxes, and those with similar expression profiles are grouped together. This analysis shows that while miR-17-5p targets both pro- and anti-proliferative targets, pro-proliferative targets are specifically up-regulated in the HEK293T-17-5p network. **(b) **A proposed model depicting the ability of miR-17-5p to act as both a tumor suppressor and an oncogene, depending on the cellular context, and using the same color and shape schema as above. In a situation where pro-proliferative genes dominate (left), suppression of anti-proliferative targets is reinforced by removal of self-regulatory signals and increased suppression by pro-proliferative regulators. These signals combine and lead to a net proliferative (oncogenic) outcome. In situations where anti-proliferative genes dominate (right), suppression of pro-proliferative signals is reinforced, leading to a net anti-proliferative signal. In this case, removal of miR-17-5p results in a pro-proliferative signal - a classic tumor suppressor outcome.

Consistent with the miR-17-5p oncogenic potential of constitutive expression, most anti-proliferative targets (12 in total) displayed either down-regulation or little change to mRNA levels, while pro-proliferative target mRNAs (8 in total) displayed marked increases in expression of 5-fold or greater in stable cell lines, leading to a net pro-proliferative signal (Figure 7a).

## Discussion

Understanding the mechanism through which the miR-17-92 locus is able to promote cellular proliferation and tumorigenesis in multiple cell lines and tissues is essential if miRNAs from this polycistron are to be seriously considered as therapeutic targets. Here we have demonstrated the ability of a single miRNA from this locus, miR-17-5p, to drive a hyper-proliferative phenotype, acting to suppress the G1/S cell cycle checkpoint and dramatically increase the proliferation rate of the cell. We reveal that miR-17-5p targets a large genetic network of interacting proteins that act co-ordinately to control the transition from G1 to S phase. Rather than "fine tuning" cell cycle progression as previously suggested [26], we propose that this coordinated targeting allows miR-17-5p to efficiently de-couple negative regulators of the MAPK signaling cascade, promoting growth in HEK293T cells (Figure 7a).

If the primary function of miR-17-5p is to interfere with cell cycle regulation, then we might expect: that the primary transcript encoding miR-17-5p and the mature miRNA are cell cycle regulated; and that its maximal expression will be at a time prior to the mature miRNAs maximal activity. For example, the G1 specific proteins CCND1 and CCND2 have their peak mRNA expression in G2/M [27], which allows time for transport and translation before the mature protein is required. Similarly, the process of miRNA maturation involves multiple processing and transportation steps, and non-coordinated dynamic expression of miRNAs from the miR-17-92 cluster suggests that this process is highly regulated [6,16,20]. Indeed, we find that the locus is cell cycle regulated, and the maximal expression of mature miR-17-5p is in the G2/M phase of HeLa cells. This timing allows translational suppression of proteins that affect the activity of proteins that start to accumulate in this phase, and confirms a likely functional action upon the G1/S transition boundary.

The miR-17-92 locus is known to be regulated by the MYC oncogene, and the E2F family of transcription factors [5,17,28]. Phase-enriched expression of miR-17-92 was not previously observed in serum stimulated primary fibroblasts [5]; however, the typical degree of synchrony achievable with this cell type (60-80%) may have prevented detection of phase-enrichment within this experiment [27,29,30]. In our study, we observed the lowest expression of this gene during S-phase. Interestingly, miR-17-92 expression also decreased (non-significantly) at 16 hours in synchronized fibroblasts [5], which is a time-point consistent with the induction of S-phase in this cell type [31]. Although not tested here, it seems likely that any periodicity of the miR-17-92 locus would be driven by the cell-cycle regulated E2F family of transcription factors [27] rather than the transiently expressed MYC [32].

Regulation of the G1/S transition by miRNAs has previously been reported as essential for germ line stem cell division in *Drosophila melanogaster*, allowing stem cells to proliferate in an environment where most other cells are quiescent [33]. Interestingly, the *Drosophila *bypass appears to be mediated through the Dap protein, an orthologue of human CDKN1A. In our study, CDKN1A was found to be a possible target of miR-17-5p directly, but more importantly was central to our genetic network, with at least five miR-17-5p targets acting to influence the levels of this protein (Figure 7). Consistent with a similar endogenous function in vertebrates, the miR-17-92 locus is highly expressed in mouse embryonic stem cells and chicken embryos, with expression levels decreasing during development and differentiation [34,35]. Two recent studies also show that expression of this miRNA is reduced when cells exit the cell cycle. The miR-17-92 cluster of miRNAs is down-regulated in female primordial germ cells as they enter meiosis (and exit from their normal, rapidly proliferating state) [36]. In B cells, expression of miR-17-5p is critical for early B cell development, but expression is greatly reduced upon B cell maturation, also marked by exit from the cell cycle [37].

Whilst miR-17-5p is capable of interacting with a number of known promoters of cellular proliferation, the mRNA levels of these genes in the stable system are greatly increased, leading to counteraction of the activity of miR-17-5p translational repression. This discrepancy cannot be explained by factors that interfere with miRNA binding, as the cells used to test miRNA-mRNA interactions were also used to assay endogenous mRNA and protein levels. Rather, the compensatory increase of mRNA levels is likely due to the combinatorial effect of withdrawing a number of important transcriptional regulators. This highlights the importance of considering biological phenotypes as the result of genetic networks subject to multiple layers of regulation, rather than the overly simplistic view of single molecular interactions driving phenotypes. This network model can also explain the ability of miR-17-5p to act as an oncogene or a tumor suppressor in different cellular contexts, dependant on the expression of other transcriptional regulators. In cell systems where the expression of the proliferation-promoters dominates, miR-17-5p would stabilize the pro-proliferative signal by removing proliferation-inhibitors, and increasing the mRNA levels of proliferation-promoters. Conversely, in systems where proliferation-inhibitors dominate, withdrawal of miR-17-5p would lead to increased proliferation-promoters and decreased mRNA levels of proliferation-inhibitors (Figure 7b).

We have uncovered a large genetic network in this study, although it is likely that this does not represent the complete story. Only genes known to be involved with the cell cycle were considered for this analysis, and as IPA interactions are based only on published data, little studied molecules, or molecules not previously associated with progression of the cell cycle are likely to be overlooked. Novel components of this network are likely to be identified by dual interactions with miR-17-5p and its target genes. The methods of pathway analysis presented here provide a unique and rapid approach to the discovery of miRNA function, regardless of how few miRNA-mRNA interactions have been previously described.

## Conclusion

We find that miR-17-92 is a cell cycle regulated locus, and a single miRNA from this cluster, miR-17-5p, is sufficient to drive a hyper-proliferative phenotype in HEK293T cells. This miRNA acts to suppress the G1/S cell cycle checkpoint and dramatically increase the proliferation rate of the cell by targeting a large genetic network of interacting proteins. This coordinated targeting allows miR-17-5p to efficiently de-couple negative regulators of the MAPK signaling cascade, promoting growth in HEK293T cells. Targeting of both proliferation-promoters and proliferation-inhibitors allows this miRNA to act as both a tumor suppressor and an oncogene in different cellular contexts.

## Materials and methods

### Network and functional analyses

miRNA-mRNA interactions were predicted by PicTar [38]. Sets of 1,000 random genes were generated using the random gene selection tool [39]. Lists of GenBank gene identifiers were uploaded into IPA [40]. Each gene identifier was mapped to its corresponding gene object in the Ingenuity Pathways Knowledge Base. The Functional Analysis tool identified the biological functions that were most represented in data sets uploaded. Although IPA uses a Fischer's exact test to calculate a *p*-value, we did not use this to determine the significance of this enrichment. Instead, the mean and standard deviation of the negative log of the *p*-values derived from random gene sets was calculated for each biological function tested. A biological function was considered to be significantly enriched if the negative log of the *p*-value was more than four standard deviations away from the mean for that function.

### Plasmid construction

Predicted target sites of miR-17-5p were cloned into the *Spe*I and *Hin*dIII sites of pMIR-REPORT Luciferase (Ambion, Austin, TX, USA). Synthetic oligos corresponding to 60 nucleotides surrounding the target sequence were annealed before ligation into the pMIR plasmid. To create plasmids expressing miR-17-5p, synthetic oligos were annealed and ligated into the *Bam*H1 and *Hin*dIII sites of pSilencer 4.1 CMV-puro (Ambion). A list of all primers used is available in Additional data file 4. All constructs were verified by sequencing.

### Selection of stable pSilencer cell lines

HEK293T cells were maintained in DMEM (Invitrogen, Mount Waverley, VIC, Australia) containing 10% (v/v) fetal calf serum, in a 5% CO_2 _atmosphere at 37°C. Cells were transfected with either pSilencer-17-5p (HEK293T-17-5p) or the parent pSilencer plasmid (HEK293T-control) using Effectene (Qiagen, Doncaster, VIC, Australia) according to manufacturer's instructions. After 24 h, puromycin selection began at 500 ng/ml. After one week, selection pressure was increased to 1 μg/ml puromycin. Individual colonies were selected two weeks post-transfection, and tested for miRNA activity (Additional data file 2).

### MTT cell proliferation assays

HEK293T cells were transiently transfected with either 10 or 50 nM of the appropriate pre-miR miRNA precursor (Ambion), using HiPerfect (Qiagen) according to the manufacturer's instructions. Stable pSilencer cell lines were plated at 1 × 10^4 ^cells per well. MTT (3-[4,5-dimethylthiazol-2-yl]-2,5-diphenyl tetrazolium bromide) activity was assayed using a Cell Growth Determination Kit (Sigma-Aldrich, Castle Hill, NSW, Australia) according to the manufacturer's instructions and detected on a PowerWave XS spectrophotometer (BioTek, Winooski, VT, USA). Doubling times were calculated from best-fit curves generated in GraphPad Prism 4 (Graphpad Software, La Jolla, CA, USA).

### Cell cycle blocks and synchronization

#### Thymidine

HEK293T-17-5p and HEK293T-control cells were treated with 2.5 mM thymidine (Sigma-Aldrich) for 16 h, released into fresh media for 8 h, and treated again with 2.5 mM thymidine for another 16 h.

#### Hydroxyurea

HEK293T-17-5p and HEK293T-control cells were incubated with 2 mM hydroxyurea (Sigma-Aldrich) for 16 h.

#### Serum starvation

HEK293T-17-5p and HEK293T-control cells were incubated in DMEM with no fetal calf serum for 48 h. HeLa cells were synchronized by incubation for 18 h with 2.5 mM thymidine (Sigma-Aldrich), released into fresh media for 8 h, and treated again with 2.5 mM thymidine for another 18 h. To obtain synchronized populations, these cells were then released for 0 h (S phase), 8 h (G2/M), and 14 h (G1/G0). Chemically synchronized populations were verified by flow cytometry.

### Flow cytometry

All cells were harvested and fixed in 70% ethanol at -20°C overnight, then resuspended in buffer (5 mM EDTA, PBS, pH 7.4) approximately 1 h prior to analysis. DNA was stained using 40 μg/ml propidium iodide (Sigma-Aldrich), and RNA was removed using 400 μg/ml RNase A (Sigma-Aldrich). Cells were filtered through 35 μm cell strainer mesh (Becton Dickinson, North Ryde, NSW, Australia) and analyzed on Becton Dickinson LSR II flow cytometer fitted with 488 nm laser. Cell data were gated using WinList v6.0 and analyzed in Modfit LT v3.0, both programs from Verity Software House (Topsham, ME, USA).

### Luciferase assays of potential miRNA binding sites

HEK293T-17-5p#1 cells were co-transfected with 50 ng of a pMIR-REPORT Luciferase construct, 50 ng of pMIR-REPORT β-galactosidase (Ambion), and 10 pmol of 2'-O-Me ASOs. Anti-17-5p and control sequences were previously described [5]. After transfection, cells were incubated for 22-24 h prior to assaying. For transient expression assays with dsRNA, HEK293T cells were transfected as above, substituting either 10 or 50 nM of the appropriate pre-miR miRNA precursor (Ambion) for ASOs. After transfection, cells were incubated for 42 h prior to harvesting. Luciferase activity was assayed using the Luciferase Assay System (Promega Corporation, Alexandria NSW, Australia), and detected on a Wallac 1420 luminometer (Perkin Elmer, Waltham, MA, USA). β-Galactosidase activity was determined using the β-Galactosidase Enzyme Assay System (Promega), and detected on a PowerWave XS spectrophotometer (BioTek). Luciferase activity was normalized to β-galactosidase activity in each well. Assays were conducted in triplicate, and independently repeated three times.

### RNA purification and qRT-PCR analyses

Total RNA was purified from cell pellets using either an RNeasy Mini Kit (Qiagen), or a miRNeasy Mini Kit (Qiagen), and in both cases RNA integrity was assessed using an Agilent Bioanalyzer 2100. For mRNA, cDNA was synthesized using SuperScript III (Invitrogen), and qRT-PCR was performed using SYBR green PCR master-mix (Applied Biosystems, Scoresby, VIC, Australia). For mature miRNA, cDNA was synthesized using a Taqman MicroRNA RT Kit (Applied Biosystems), and qRT-PCR was performed using a miR-17-5p MicroRNA Taqman assay (Applied Biosystems). All RT-PCR was performed on an Applied Biosystems 7000 Sequence Detection System. Control reactions without reverse transcriptase were performed to check for DNA contamination. Details of all primers used are available in Additional data file 4.

### Antibodies and immunoblots

Cells were washed twice with PBS and resuspended in sample buffer (50 mM Tris-Cl pH 6.8; 100 mM dithiothreitol; 2% (w/v) SDS; 10% (v/v) glycerol), and allowed to lyse on ice for 10 minutes. After lysis, samples were cleared by centrifugation at 10,000 × *g *for 10 minutes at 4°C. Samples were analyzed by immunoblot using standard procedures [41]. Rabbit anti-actin (Sigma-Aldrich) was used at 1 in 200. Rabbit anti-cyclin D1 (SP4; Neomarkers Inc, Freemont, CA, USA), rabbit anti-SAPK/JNK (56G8, Cell Signaling Technology, Boston, MA, USA), and mouse anti-Rb2 (10/Rb2; Becton Dickinson) were all used at 1 in 500. Goat-anti-mouse-HRP and goat-anti-rabbit-HRP (Bio-Rad, Gladesville, NSW, Australia) were used at 1 in 2,000, and detected using the SuperSignal West Pico Chemiluminescent Substrate (Pierce Biotechnology, Murarrie, QLD, Australia).

## Abbreviations

ASO, 2'-O-Methyl antisense oligoribonucleotide; DMEM, Dulbecco's modified Eagle's media; dsRNA, double-stranded RNA; IPA, Ingenuity Pathways Analysis; MAPK, mitogen activated protein kinase; miRNA, microRNA; MTT, 3-[4,5-dimethylthiazol-2-yl]-2,5-diphenyl tetrazolium bromide; PBS, phosphate-buffered saline; qRT-PCR, quantitative real time PCR; UTR, untranslated region.

## Authors' contributions

NC and SG conceived and coordinated the study, and drafted the manuscript. NC, SG, and AF participated in experimental design and data analysis. NC analyzed miRNA function, and performed MTT assays. BG synchronized the HeLa cells. MB and BG generated the DNA profiles. SW, MB, GK, and WC performed qRT-PCR. AS, SW, WC, and NC cloned miRNA target sites. AS and NC generated stable cell lines, and performed luciferase and β-galactosidase assays. AS, SW, and NC performed western blots. All authors read and approved the final manuscript.

## Additional data files

The following additional data are available with the online version of this paper. Additional data file 1 is a table listing the G1/S associated mRNAs predicted to be targets of miR-17-5p. Additional data file 2 is a figure showing the validation of miR-17-5p activity in stable HEK293T cell lines over-expressing miR-17-5p. Additional data file 3 is a figure depicting the interactions between miR-17-5p targets and cell cycle components. An interactive version of this figure where literature support and gene/protein information can be viewed through IPA is available [24]. Additional data file 4 is table listing all primers used in this study.

## Supplementary Material

Additional data file 1 G1/S associated mRNAs predicted to be targets of miR-17-5p. Click here for file

Additional data file 2 Validation of miR-17-5p activity in stable HEK293T cell lines over-expressing miR-17-5p. Click here for file

Additional data file 3 An interactive version of this figure where literature support and gene/protein information can be viewed through IPA is available [24]. Click here for file

Additional data file 4 Primers used in this study. Click here for file
