# Supplementary material for: The miR-17-5p microRNA is a key regulator of the G1/S phase cell cycle transition
Source: Genome Biol. 2008 Aug 14;9(8):R127. doi: 10.1186/gb-2008-9-8-r127 (PMC2575517; doi:10.1186/gb-2008-9-8-r127)
Supplement: Additional data file 1 — G1/S associated mRNAs predicted to be targets of miR-17-5p. [file gb-2008-9-8-r127-S1.pdf]

Supplementary Table S1. Genes in the miR-17-5p cell cycle network. For each predicted target, the HUGO symbol and GenBank accession number are listed with their PicTar score for hsa-mir-17-5p interaction, the rank in the list of all PicTar predictions for that miRNA, and the number of sites within the transcript that miR-17-5p is predicted to bind to.

HUGO Gene Symbol	GenBank	Description	PicTar Score	PicTar Rank	# sites
APBB2	NM_173075	amyloid beta (A4) precursor protein-binding, family B, member 2 (Fe65-like)	1.82	605	3
APP	NM_000484	amyloid beta (A4) precursor protein (peptidase nexin-II, Alzheimer disease)	2.35	478	4
BCL2L11	NM_006538	BCL2-like 11 (apoptosis facilitator)	4.05	154	5
CCND1	NM_053056	cyclin D1	2.3	488	4
CCND2	NM_001759	cyclin D2	1.45	671	2
CCNG2	NM_004354	cyclin G2	2.63	413	2
CDKN1A	NM_000389	cyclin-dependent kinase inhibitor 1A (p21, Cip1)	4.19	138	3
CRK	NM_016823	v-crk sarcoma virus CT10 oncogene homolog (avian)	2.79	371	1
CUL3	NM_003590	cullin 3	1.19	699	4
DMTF1	NM_021145	cyclin D binding myb-like transcription factor 1	3.85	181	2
E2F1	NM_005225	E2F transcription factor 1	6.57	21	3
E2F3	NM_001949	E2F transcription factor 3	1.24	696	2
E2F5	NM_001951	E2F transcription factor 5, p130-binding	2.85	360	2
EREG	NM_001432	Epiregulin	3.76	193	4
FOXO1A	NM_002015	forkhead box O1A (rhabdomyosarcoma)	2.89	345	5
GAB1	NM_002039	GRB2-associated binding protein 1	2.56	426	1
HAS2	NM_005328	hyaluronan synthase 2	2.66	409	1
HDAC4	NM_006037	histone deacetylase 4	4.43	124	3
HIF1A	NM_001530	hypoxia-inducible factor 1, alpha subunit (basic helix-loop-helix transcription factor)	4.86	75	2
IRF1	NM_002198	interferon regulatory factor 1	2.71	388	1
KHDRBS1	NM_006559	KH domain containing, RNA binding, signal transduction associated 1	2.46	445	2
KPNA2	NM_002266	karyopherin alpha 2 (RAG cohort 1, importin alpha 1)	3.67	211	1
MAP3K8	NM_005204	mitogen-activated protein	2.64	412	1

		kinase kinase kinase 8			
MAPK9	NM_002752	mitogen-activated protein kinase 9	2.98	322	1
MYCN	NM_005378	v-myc myelocytomatosis viral related oncogene, neuroblastoma derived (avian)	6.36	27	3
NCOA3	NM_006534	nuclear receptor coactivator 3	3.71	202	4
NR4A3	NM_006981	nuclear receptor subfamily 4, group A, member 3	5.38	53	3
PCAF	NM_003884	p300/CBP-associated factor	3.03	313	2
PDGFRA	NM_006206	platelet-derived growth factor receptor, alpha polypeptide	2.36	475	3
PKD1	NM_000296	polycystic kidney disease 1 (autosomal dominant)	2.79	370	1
PKD2	NM_000297	polycystic kidney disease 2 (autosomal dominant)	4.01	158	4
PPARA	NM_001001928	peroxisome proliferative activated receptor, alpha	3.44	239	8
PTEN	NM_000314	phosphatase and tensin homolog	n/a	n/a	1
RB1	NM_000321	retinoblastoma 1 (including osteosarcoma)	3.97	165	2
RB1CC1	NM_014781	RB1-inducible coiled-coil 1	n/a	n/a	2
RBBP7	NM_002893	retinoblastoma binding protein 7	3.58	222	1
RBL1	NM_002895	retinoblastoma-like 1 (p107)	3.87	180	2
RBL2	NM_005611	retinoblastoma-like 2 (p130)	5.92	36	3
RNF111	NM_017610	ring finger protein 111	n/a	n/a	1
SMAD7	NM_005904	SMAD family member 7	n/a	n/a	1
STAT3	NM_139276	signal transducer and activator of transcription 3 (acute-phase response factor)	4.09	149	4
TIMP2	NM_003255	TIMP metallopeptidase inhibitor 2	n/a	n/a	4
TP53INP1	NM_033285	tumor protein p53 inducible nuclear protein 1	3.69	207	7
TSG101	NM_006292	tumor susceptibility gene 101	3.68	210	1
TXNIP	NM_006472	Thioredoxin interacting protein	5.72	44	3
WEE1	NM_003390	WEE1 homolog (S. pombe)	2.65	411	1
