# Supplementary material for: The miR-17-5p microRNA is a key regulator of the G1/S phase cell cycle transition
Source: Genome Biol. 2008 Aug 14;9(8):R127. doi: 10.1186/gb-2008-9-8-r127 (PMC2575517; doi:10.1186/gb-2008-9-8-r127)
Supplement: Additional data file 2 — Validation of miR-17-5p activity in stable HEK293T cell lines over-expressing miR-17-5p. [file gb-2008-9-8-r127-S2.pdf]

Figure S2.

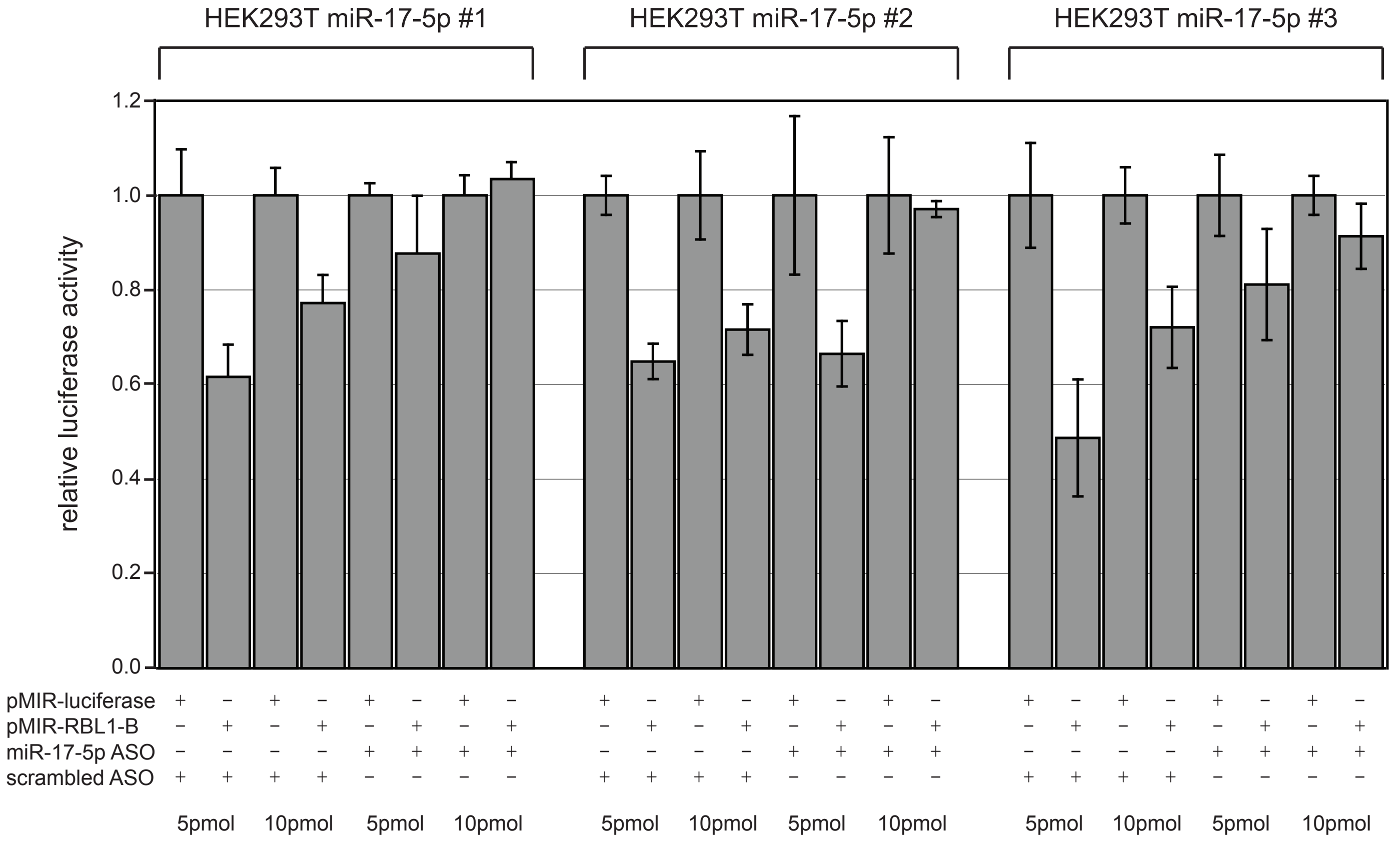

Supplementary Figure S2. Validation of miR-17-5p activity in HEK293T-17-5p stable cell lines. Stable cell lines were co-transfected with the reporter pMIR-RBL1-B plasmid and with either miR-17-5p or scrambled 2'-O-Me anti-sense oligos (ASOs). Rescue of the luciferase activity by miR-17-5p ASO was compared to the luciferase activity of the parental plasmid containing no miR-17-5p binding sites. Plotted are the means and s.e.ms of relative luciferase activity.
