# Supplementary figures and images for: The miR-17-5p microRNA is a key regulator of the G1/S phase cell cycle transition
Source: Genome Biol. 2008 Aug 14;9(8):R127. doi: 10.1186/gb-2008-9-8-r127 (PMC2575517; doi:10.1186/gb-2008-9-8-r127)

Extracellular Space

Plasma Membrane

Cytoplasm

Nucleus

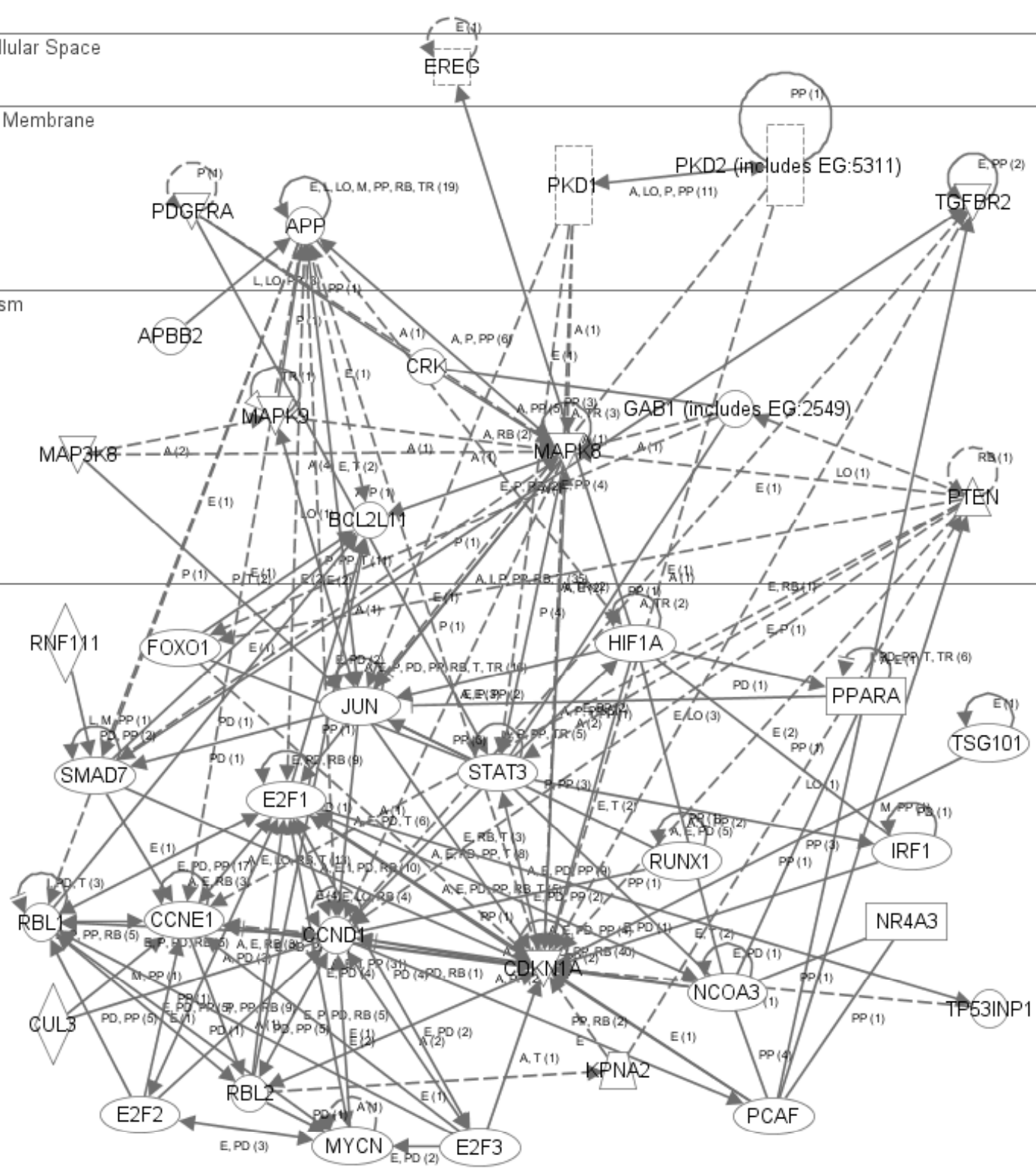

Supplement: Additional data file 3 — An interactive version of this figure where literature support and gene/protein information can be viewed through IPA is available [24]. [file gb-2008-9-8-r127-S3.pdf]
