# Supplementary material for: The miR-17-5p microRNA is a key regulator of the G1/S phase cell cycle transition
Source: Genome Biol. 2008 Aug 14;9(8):R127. doi: 10.1186/gb-2008-9-8-r127 (PMC2575517; doi:10.1186/gb-2008-9-8-r127)
Supplement: Additional data file 4 — Primers used in this study. [file gb-2008-9-8-r127-S4.pdf]

Supplementary Table S4. A list of all primers used in this study. Primers used for qRT-PCR are suffixed with –qRT-[direction]. Remaining primers were used to recreate binding sites of miRNAs for cloning into pMIR-REPORT Luciferase.

Primer Name	Primer Sequence (5' -> 3')
C13ORF25-qRT-L1	CGGCTACTCCTCCTGTCATAC
C13ORF25-qRT-R1	CCACTTAGCAAATACCATTCCA
BCL2L11-qRT-L1	GGCGTATTGGAGACGAGTTT
BCL2L11-qRT-R1	GTCAATGCATTCTCCACACC
CCNG2-qRT-L1	ATAGTGTTCCTGAGCTGCCA
CCNG2-qRT-R1	TGATCACTGGGAGGAGAGC
CDKN1A-qRT-L1	GGCTGATCTTCTCCAAGAGG
CDKN1A-qRT-R1	AAGATGTAGAGCGGGCCTT
E2F3-qRT-L1	GCTGCAGTCTGTCTGAGGAT
E2F3-qRT-R1	AACAGTTTGAGGTCCAGGGT
E2F5-qRT-L1	GGAGGTACCCATTCCAGAAA
E2F5-qRT-R1	AACCACGGGCTTAGATGAAC
GAB1-qRT-L1	CAATGAATCCCAATTCACCA
GAB1-qRT-R1	CAGGAGGAGGAACTTGCATT
IRF1-qRT-L1	CTCAGCTGTGCGAGTGTACC
IRF1-qRT-R1	GAGAAGGTATCAGGGCTGGA
MAP3K8-qRT-L1	AGGCTGCTGAGTAGGAAGGA
MAP3K8-qRT-R1	GATTGAAGTAGCCAGCCAGA
MAPK9-qRT-L1	AGCTGGTGAAAGGTTGTGTG
MAPK9-qRT-R1	TTCCTCACAGTTGGCTGAAG
MYCN-qRT-L1	ACGTGGTCACTGTGGAGAAG
MYCN-qRT-R1	CATCGTTTGAGGATCAGCTC
NR4A3-qRT-L1	GCCCTGGTAGAACTGAGGAA
NR4A3-qRT-R1	GGAAGGTGGAGACACCAAGT
PCAF-qRT-L1	CCACAGTTCTGCGACAGTCT
PCAF-qRT-R1	TAACAGTGAAGACCGAGCGA
PDK2-qRT-L1	CTCCTTATTGGATGGGCTGT
PDK2-qRT-R1	ATTTCTGACTCGGAGTTGCC
PTEN-qRT-L1	TCTGGATTATAGACCAGTGGCA
PTEN-qRT-R1	CTTTAGCTGGCAGACCACAA
TSG101-qRT-L1	GGGCCACCAAATACTTCCTA
TSG101-qRT-R1	TGTTGTGGCAGGATATGGAC
CCND2-qRT-L1	GTAATGTGGCCTTGGCATTT
CCND2-qRT-R1	CTATCGCTCGGGAACAATTT
FOXO1A-qRT-L1	TACAGGATGTTTGCCCAATG
FOXO1A-qRT-R1	GAAAGCACACCAGGATCTGA
WEE1-qRT-L1	TCTTGGGCATGTAACAAGGA
WEE1-qRT-R1	ACATACCACTGTGAGGGCAA
CCND1-qRT-L1	AAGAGTGTGGAGGCTGACG
CCND1-qRT-R1	ACGGTAGCAGCGCAATAAG
PDK1-qRT-L1	GTGTGCCTGGTGACCTACAT

PDK1-qRT-R1	GCCTGTCTTGACGAGGATCT
RB1-qRT-L1	AACCTCAGCCTTCCAGACC
RB1-qRT-R1	TCTGGGTGCTCAGACAGAAG
HIF1A-qRT-L1	TGCTGATTTGTGAACCCATT
HIF1A-qRT-R1	TCTGGCTCATATCCCATCAA
RBL2-qRT-L1	CAGCAGTGATAGCAGAAGCC
RBL2-qRT-R1	GTAGGTGTGGGAGGAGCACT
APP-qRT-L1	TTCCCTACCGCTGCTTAGTT
APP-qRT-R1	TGAGTTTCGCAAACATCCAT
PPARA-qRT-L1	TGGAGCATTGAACATCGAAT
PPARA-qRT-R1	GGTCGCACTTGTCATACACC
RBL1-qRT-L1	TGTCTCCTCTAATGCACCCA
RBL1-qRT-R1	GCGTTCATGGACAGAAATTG
STAT3-qRT-L1	GGGAAGAATCACGCCTTCTA
STAT3-qRT-R1	CACTCTCTTCCGGACATCCT
CRK-qRT-L1	TTCATTGCCTGCTTTACTGG
CRK-qRT-R1	AAAGAGGGCTCGCACATACT
E2F1-qRT-L1	CATCCCAGGAGGTCACTTCT
E2F1-qRT-R1	GTTCTTGCTCCAGGCTGAGT
NCOA3-qRT-L1	CCCAAACCAGCAGAATATCA
NCOA3-qRT-R1	AGAAGATGCCATGGGAGAGT
APBB2-A-F	CTAGggactgtttaacaagtttcccaaaacaGCATTTTcctgctccttcgtatgtaggtgagaa
APBB2-A-R	AGCTttctcacctacatacgaaggagcaggAAAATGCtgttttgggaaacttgttaaacagtcc
APBB2-B-F	CTAGtttaaaagagattaataaaatcataatGCATTTTgggtgggacatatttcaaacttctgc
APBB2-B-R	AGCTgcagaagtttgaaatatgtcccacccAAAATGCattatgattttattaatctcttttaaa
APBB2-C-F	CTAGtgtcacaagcaacctttaagtcctacaGCACTTTgccctgttttcaacattggagtaggc
APBB2-C-R	AGCTgcctactccaatgttgaaaacagggcAAAGTGCtgtaggacttaaaggttgcttgtgaca
APP-A-F	CTAGctttagagagattttttttccatgactGCATTTTactgtacagattgctgcttctgctat
APP-A-R	AGCTatagcagaagcagcaatctgtacagtAAAATGCagtcatggaaaaaaaatctctctaaag
APP-B-F	CTAGaaagaaaagaatccctgttcattgtaaGCACTTTtacggggcgggtggggaggggtgctc
APP-B-R	AGCTgagcacccctccccacccgccccgtaAAAGTGCttacaatgaacagggattcttttcttt
APP-C-F	CTAGcctaagtattcctttcctgatcactatGCATTTTaaagttaaacatttttaagtatttca
APP-C-R	AGCTtgaaatacttaaaaatgtttaactttAAAATGCatagtgatcaggaaaggaatacttagg
APP-D-F	CTAGatcagtaatgtattctatctctctttaCATTTTGgtctctatactacattattaatgggt
APP-D-R	AGCTacccattaataatgtagtatagagacCAAAATGtaaagagagatagaatacattactgat
BCL2L11-A-F	CTAGctttgcggagccgagataccatgcagaCATTTTGcttgttcaaaccaacaagacccagca
BCL2L11-A-R	AGCTtgctgggtcttgttggtttgaacaagCAAAATGtctgcatggtatctcggctccgcaaag
BCL2L11-B-F	CTAGttgcaggctttcccatggtcacaggatGCACTGTcagcatcaggtcccagagggccaccg
BCL2L11-B-R	AGCTcggtggccctctgggacctgatgctgACAGTGCatcctgtgaccatgggaaagcctgcaa
BCL2L11-C-F	CTAGttctgatggccatttgcttggcctcctGCATTTTagtccaactcacagtccactagcttc
BCL2L11-C-R	AGCTgaagctagtggactgtgagttggactAAAATGCaggaggccaagcaaatggccatcagaa
BCL2L11-D-F	CTAGagtccactagcttcactcctttaaattCACTTTGaaacaggcctcatcccacttccacca
BCL2L11-D-R	AGCTtggtggaagtgggatgaggcctgtttCAAAGTGaatttaaaggagtgaagctagtggact
BCL2L11-E-F	CTAGtactcacgtgccagtctcctgactagaGCACTTTactctgtttcctcagccctgcagccc
BCL2L11-E-R	AGCTgggctgcagggctgaggaaacagagtAAAGTGCtctagtcaggagactggcacgtgagta
CCND1-A-F	CTAGtctgtctgaaccacgcgggggccttgaGGGACGCtttgtctgtcgtgatggggcaagggc
CCND1-A-R	AGCTgcccttgccccatcacgacagacaaaGCGTCCCtcaaggcccccgcgtggttcagacaga
CCND1-B-F	CTAGctcccctgacagtccctcctctccggaGCATTTTgataccagaagggaaagcttcattct
CCND1-B-R	AGCTagaatgaagctttcccttctggtatcAAAATGCtccggagaggagggactgtcaggggag
CCND1-C-F	CTAGcatattctaaaaccattccatttccaaGCACTTTcagtccaataggtgtaggaaatagcg

CCND1-C-R	AGCTcgctatttcctacacctattggactgAAAGTGCttggaaatggaatggttttagaatatg
CCND1-D-F	CTAGcctcttctcttccctgcgcctgtgatgCTGGGCActtcatctgatcgggggcgtagcatc
CCND1-D-R	AGCTgatgctacgcccccgatcagatgaagTGCCCAGcatcacaggcgcagggaagagaagagg
CCND2-A-F	CTAGattgttataaaccattccattcgaaaaGCACTTTgaaaaattgttcccgagcgatagatg
CCND2-A-R	AGCTcatctatcgctcgggaacaatttttcAAAGTGCttttcgaatggaatggtttataacaat
CCND2-B-F	CTAGagatagatggctgaacatcagggtgtgGCATTTTgttcccttttccgttttttttttt
CCND2-B-R	AGCTaaaaaaaaaaaaacggaaaagggaacAAAATGCcacaccctgatgttcagccatctatct
CCNG2-A-F	CTAGtgttgatctggtgtagtatattttatcGCATTTTcttatattaaaaaatgtctgcatgat
CCNG2-A-R	AGCTatcatgcagacatttttaatataagAAAATGCgataaaatatactacaccagatcaaca
CCNG2-B-F	CTAGaaaccattaacagtactttagacattgGCACTTTatttttctcgtagatctttagctact
CCNG2-B-R	AGCTagtagctaaagatctacgagaaaaatAAAGTGCcaatgtctaaagtactgttaatggttt
CDKN1A-A-F	CTAGtgaatgagaggttcctaagagtgctggGCATTTTtattttatgaaatactatttaaagcc
CDKN1A-A-R	AGCTggctttaaatagtatttcataaaataAAAATGCccagcactcttaggaacctctcattca
CDKN1A-B-F	CTAGctttttcatttgagaagtaaacagatgGCACTTTgaaggggcctcaccgagtgggggcat
CDKN1A-B-R	AGCTatgcccccactcggtgaggccccttcAAAGTGCcatctgtttacttctcaaatgaaaaag
CDKN1A-C-F	CTAGatccaccccatccctccccagttcattGCACTTTgattagcagcggaacaaggagtcaga
CDKN1A-C-R	AGCTtctgactccttgttccgctgctaatcAAAGTGCaatgaactggggagggatggggtggat
CRK-A-F	CTAGgcgcggtggctcacgtctgtaatcccaGCACTTTgggaggccgaggcgggtggatcatga
CRK-A-R	AGCTtcatgatccacccgcctcggcctcccAAAGTGCtgggattacagacgtgagccaccgcgc
CRK-B-F	CTAGttccaggtgaaaagcaggatgtaccgaGCACTTTattcagtgcatagctttaagccagtg
CRK-B-R	AGCTcactggcttaaagctatgcactgaatAAAGTGCtcggtacatcctgcttttcacctggaa
CUL3-A-F	CTAGgaatgtgtccattgaaaagaaaataggCGCTTTGtctcctacaatcttagagaaagtttt
CUL3-A-R	AGCTaaaactttctctaagattgtaggagaCAAAGCGcctattttcttttcaatggacacattc
CUL3-B-F	CTAGctctgtaagaatccttctcatagtgaaGCACTGTtgatgttttgttcaccggatatgttg
CUL3-B-R	AGCTcaacatatccggtgaacaaaacatcaACAGTGCttcactatgagaaggattcttacagag
CUL3-C-F	CTAGtatcttcaattttatttaattttgtttCATTTTGttttcataagacaatgtttcagatat
CUL3-C-R	AGCTatatctgaaacattgtcttatgaaaaCAAAATGaaacaaaattaaataaaattgaagata
CUL3-D-F	CTAGtgtaaatgtgttttgtagtgttgcttaGCACTTTgcgcattgtgtaagttgggtaacaaa
CUL3-D-R	AGCTtttgttacccaacttacacaatgcgcAAAGTGCtaagcaacactacaaaacacatttaca
DMTF1-A-F	CTAGgtacagtttctctaaagatcagacatgGCACTGTctcctctcaagcctggttgtagttca
DMTF1-A-R	AGCTtgaactacaaccaggcttgagaggagACAGTGCcatgtctgatctttagagaaactgtac
DMTF1-B-F	CTAGaaccactgtcattagtttacaaagttaGCACTTTgaagtaaaactaaatgaggaaggaag
DMTF1-B-R	AGCTcttccttcctcatttagttttacttcAAAGTGCtaactttgtaaactaatgacagtggtt
E2F1-A-F	CTAGccactgctctgccccaccctccaatctGCACTTTgatttgcttcctaacagctctgttcc
E2F1-A-R	AGCTggaacagagctgttaggaagcaaatcAAAGTGCagattggagggtggggcagagcagtgg
E2F1-B-F	CTAGgctggctgggcgtgtaggacggtgagaGCACTTCtgtcttaaaggtttttctgattgaa
E2F1-B-R	AGCTttcaatcagaaaaacctttaagacaGAAGTGCtctcaccgtcctacacgcccagccagc
E2F1-C-F	CTAGatgtgtgcgcgtgggggggctctaactGCACTTTcggcccttttgctctgggggtcccac
E2F1-C-R	AGCTgtgggacccccagagcaaaagggccgAAAGTGCagttagagccccccacgcgcacacat
E2F3-A-F	CTAGgctttgtgttaagtgcctactggaaatGCACTGTggggtttttcctgtatgggaaacca
E2F3-A-R	AGCTtggtttcccatacaggaaaaaccccACAGTGCatttccagtaggcacttaacacaaagc
E2F3-B-F	CTAGgtggtggggtcaagacagatgacaccaGCACTTTaaactctttgtgtgggtatgcgtggg
E2F3-B-R	AGCTcccacgcatacccacacaaagagtttAAAGTGCtggtgtcatctgtcttgaccccaccac
E2F5-A-F	CTAGttctgttttagcactttaagtttatcaCATTTTGttgacttctgacattccactttccta
E2F5-A-R	AGCTtaggaaagtggaatgtcagaagtcaaCAAAATGtgataaacttaaagtgctaaaacagaa
EREG-A-F	CTAGgctattgcataaggagccactgctgccACCACTTttggattttatgggaggctccttcat
EREG-A-R	AGCTatgaaggagcctcccataaaatccaaAAGTGGTggcagcagtggctccttatgcaatagc
EREG-B-F	CTAGatgcagtttttaaaaacctgtatctgaCCCACTTtgtaatttttgctccaatatccattc
EREG-B-R	AGCTgaatggatattggagcaaaaattacaAAGTGGGtcagatacaggtttttaaaaactgcat
EREG-C-F	CTAGatttcttttattttgcactctgtaaTTGCACTttttaagtttgaagagccattttggt

EREG-C-R	AGCTaccaaaatggctcttcaaacttaaaaAGTGCAAttacagagtgcaaaataaaaagaaaat
EREG-D-F	CTAGtgtaattgcactttttaagtttgaagaGCCATTTtggtaaacggtttttattaaagatgc
EREG-D-R	AGCTgcatctttaataaaaaccgtttaccaAAATGGCtcttcaaacttaaaaagtgcaattaca
FOXO1A-A-F	CTAGattgaacagccaccactctatcatcctCATTTTGgggcagtccaagacatagctggtttt
FOXO1A-A-R	AGCTaaaaccagctatgtcttggactgcccCAAAATGaggatgatagagtggtggctgttcaat
FOXO1A-B-F	CTAGcatcacaatgaagagaacaggtgcacaGCACTGTtcctcttgtgttcttgagaaggatct
FOXO1A-B-R	AGCTagatccttctcaagaacacaagaggaACAGTGCtgtgcacctgttctcttcattgtgatg
FOXO1A-C-F	CTAGcaaacgagggattttgatccacttcacCATTTTGagttgagctttagcaaaagtttcccc
FOXO1A-C-R	AGCTggggaaacttttgctaaagctcaactCAAAATGgtgaagtggatcaaaatccctcgtttg
FOXO1A-D-F	CTAGggcaggaaagtgatgtatagttatggaCACTTTGcgtttcttatttaggataacttaata
FOXO1A-D-R	AGCTtattaagttatcctaaataagaaacgCAAAGTGtccataactatacatcactttcctgcc
FOXO1A-E-F	CTAGattttatacatgcttaactggtttgtaCACTTTGggatgctacttagtgatgtttctgac
FOXO1A-E-R	AGCTgtcagaaacatcactaagtagcatccCAAAGTGtacaaaccagttaagcatgtataaaat
FOXO1A-F-F	CTAGggtatttggggcagcattcataattttCATTTTGtattctaactggattagtactaattt
FOXO1A-F-R	AGCTaaattagtactaatccagttagaataCAAAATGaaaattatgaatgctgccccaaatacc
GAB1-A-F	CTAGacacactcgtagtattactgtatttatGCACTTTttcatctaaaacattgttctgggttt
GAB1-A-R	AGCTaaacccagaacaatgttttagatgaaAAAGTGCataaatacagtaatactacgagtgtgt
HAS2-A-F	CTAGtgaaaatgggatgaattctttgtttatGCACTTTttccttactgtgcatccgcctgaaag
HAS2-A-R	AGCTctttcaggcggatgcacagtaaggaaAAAGTGCataaacaaagaattcatcccattttca
HDAC4-A-F	CTAGaagctgagcgtgttcttagctcggcctCACTTTGtctctggcattgattaaaagtctgct
HDAC4-A-R	AGCTagcagacttttaatcaatgccagagaCAAAGTGaggccgagctaagaacacgctcagctt
HDAC4-B-F	CTAGcactttaagcagtcgtgaactgtgcgaGCACTGTggtttacaattatactttgcatcgaa
HDAC4-B-R	AGCTttcgatgcaaagtataattgtaaaccACAGTGCtcgcacagttcacgactgcttaaagtg
HDAC4-C-F	CTAGcgtcccagggctctggttctggagggcCACTTTGtcaaggtgtttcagtttttctttact
HDAC4-C-R	AGCTagtaaagaaaaactgaaacaccttgaCAAAGTGgccctccagaaccagagccctgggacg
HDAC4-D-F	CTAGgggcactggctgggagtcagcaagcgaGCACTTTatatccctttgagggaaaccctgatg
HDAC4-D-R	AGCTcatcagggtttccctcaaagggatatAAAGTGCtcgcttgctgactcccagccagtgccc
HIF1A-A-F	CTAGagagaaatcatctgatgtttctatagtCACTTTGccagctcaaaagaaaacaataccct a
HIF1A-A-R	AGCTtagggtattgttttcttttgagctggCAAAGTGactatagaaacatcagatgatttctct
HIF1A-B-F	CTAGtgctcaaaatacaatgtttgattttatGCACTTTgtcgctattaacatcctttttttcat
HIF1A-B-R	AGCTatgaaaaaaaggatgttaatagcgacAAAGTGCataaaatcaaacattgtattttgagca
IRF1-A-F	CTAGggctgaggggcttgggaaaaaaacttgGCACTTTttcgtgtggatcttgccacatttctg
IRF1-A-R	AGCTcagaaatgtggcaagatccacacgaaAAAGTGCcaagtttttttcccaagcccctcagcc
KHDRBS1-A-F	CTAGgtcctaattggatttgtaccgtcctccCATTTTGttctcggaagattaaatgctacatgt
KHDRBS1-A-R	AGCTacatgtagcatttaatcttccgagaaCAAAATGggaggacggtacaaatccaattaggac
KHDRBS1-B-F	CTAGcagctgaatgaaaaaggaatcaaaatcCACTTTGtacataagttaaagtcctaattggat
KHDRBS1-B-R	AGCTatccaattaggactttaacttatgtaCAAAGTGgattttgattcctttttcattcagctg
KPNA2-A-F	CTAGctttcttaaatgtggtttgttactgtaGCACTTTttacactgaaactatacttgaacagt
KPNA2-A-R	AGCTactgttcaagtatagtttcagtgtaaAAAGTGCtacagtaacaaaccacatttaagaaag
MAP3K8-A-F	CTAGgtgtctgctgactgtttcattcactgtGCACTTTgctcaaaattttaaaaataccaatca
MAP3K8-A-R	AGCTtgattggtatttttaaaattttgagcAAAGTGCacagtgaatgaaacagtcagcagacac
MAP3K8-B-F	CTAGaaaattcaattaggattaataaagatgGCACTTTcccgttttattccagttttataaaaa
MAP3K8-B-R	AGCTtttttataaaactggaataaaacgggAAAGTGCcatctttattaatcctaattgaatttt
MAPK9-A-F	CTAGaaatgcttgcttggacttgcccatctaGCACTTTggaaatcagtatttaaatgccaaata
MAPK9-A-R	AGCTtatttggcatttaaatactgatttccAAAGTGCtagatgggcaagtccaagcaagcattt
MYCN-A-F	CTAGtgtatatatttagtgctgcatcttataGCACTTTgaaatacctcatgtttatgaaaataa
MYCN-A-R	AGCTttattttcataaacatgaggtatttcAAAGTGCtataagatgcagcactaaatatataca
MYCN-B-F	CTAGaaactggacagtcactgccactttgcaCATTTTGatttttttttaaacaaacattgtgt
MYCN-B-R	AGCTacacaatgtttgtttaaaaaaaaaatCAAAATGtgcaaagtggcagtgactgtccagttt
NCOA3-A-F	CTAGttaaagagattatttgtttagatgtagGCATTTTaatttttaaaaattcctctaccaga

NCOA3-A-R	AGCTtctggtagaggaatttttaaaaaattAAAATGCctacatctaaacaaataatctctttaa
NCOA3-B-F	CTAGttttcacatgctaatgtgcagctgagtGCACTTTatttaaaaagaatggataaatgcaat
NCOA3-B-R	AGCTattgcatttatccattctttttaaatAAAGTGCactcagctgcacattagcatgtgaaaa
NCOA3-C-F	CTAGttttaaaaattcctctaccagaactaaGCACTTTgttaatttgggggggaaagaatagata
NCOA3-C-R	AGCTtatctattctttccccccaaattaacAAAGTGCttagttctggtagaggaatttttaaaa
NCOA3-D-F	CTAGatggtgagctgtgactgctttgctgacCATTTTGgatgtcattgtaaataaaggtttcta
NCOA3-D-R	AGCTtagaaacctttatttacaatgacatcCAAAATGgtcagcaaagcagtcacagctcaccat
NR4A3-A-F	CTAGgggttttctaagaaattgctaacaaagCACTTTTggacaatgctatcccagcaggaaaaa
NR4A3-A-R	AGCTtttttcctgctgggatagcattgtccAAAAGTGctttgttagcaatttcttagaaaaccc
NR4A3-B-F	CTAGcagtgacttttaaggcagtactgtttaGCACTTTgatattaaaattttgcttatgttttg
NR4A3-B-R	AGCTcaaaacataagcaaaattttaatatcAAAGTGCtaaacagtactgccttaaaagtcactg
NR4A3-C-F	CTAGtctgtatcaagtcaaaatatctttggcCATTTTGctaagaaacaaactttgaatgtcaaa
NR4A3-C-R	AGCTttgacattcaaagtttgtttcttagCAAAATGgccaaagatattttgacttgatacaga
PCAF-A-F	CTAGgaagagacttgtaaatgtaataattagCACTTTTgaaaaaacaaaaaacctccttttagc
PCAF-A-R	AGCTgctaaaaggaggtttttgtttttcAAAAGTGctaattattacatttacaagtctcttc
PCAF-C-F	CTAGaatcagattgtctcttctatattgaaaGCATTTTtatgttttctaatttaaaaattaata
PCAF-C-R	AGCTtattaattttaaattagaaaacataAAAATGCtttcaatatagaagagacaatctgatt
PDGFRA-A-F	CTAGtgaaccttaaaaggtactggtactataGCATTTTgctatctttttagtgttaaagagat
PDGFRA-A-R	AGCTatctctttaacactaaaaaagatagcAAAATGCtatagtaccagtaccttttaaggttca
PDGFRA-B-F	CTAGtgtacagccttattttgttggtgctttGCATTTTgatattgctgtgagccttgcatgaca
PDGFRA-B-R	AGCTtgtcatgcaaggctcacagcaatatcAAAATGCaaagcaccaacaaaataaggctgtaca
PDGFRA-C-F	CTAGgataatccccacaggcacattaactgtTGCACTTttgaatgtccaaaatttatattttag
PDGFRA-C-R	AGCTctaaaatataaattttggacattcaaAAGTGCAacagttaatgtgcctgtggggattatc
PKD1-A-F	CTAGggggcatctgtctgtctgtgggcttcaGCACTTTaaagaggctgtgtggccaaccaggac
PKD1-A-R	AGCTgtcctggttggccacacagcctctttAAAGTGCtgaagcccacagacagacagatgcccc
PKD2-A-F	CTAGgtctttgtgaccgattgctaatcttctGCACTTTaatttattttatataaactttaccca
PKD2-A-R	AGCTtgggtaaagtttatataaaataaattAAAGTGCagaagattagcaatcggtcacaaagac
PKD2-B-F	CTAGgtatgacggctcacgcctgtaatcccaGCACTTTgggaggccgaaacaggcgaatcactt
PKD2-B-R	AGCTaagtgattcgcctgtttcggcctcccAAAGTGCtgggattacaggcgtgagccgtcatac
PKD2-C-F	CTAGaagaatgttattaatgtgtaatactgaGCACTTTacttcttaataaaaacttgatatagt
PKD2-C-R	AGCTactatatcaagtttttattaagaagtAAAGTGCtcagtattacacattaataacattctt
PKD2-D-F	CTAGcttttacagaaatgttgagtaaggtgaCATTTTGagcgctaataagcaaaagagcatgca
PKD2-D-R	AGCTtgcatgctcttttgcttattagcgctCAAAATGtcaccttactcaacatttctgtaaaag
PPARA-A-F	CTAGctcctttataattctgaaaactaatcaGCACTTTttaacaatgtttataatcctataagt
PPARA-A-R	AGCTacttataggattataaacattgttaaAAAGTGCtgattagttttcagaattataaaggag
PPARA-B-F	CTAGgcacagtggctcacacatggaatcccaGCACTTTgggaggccgaggtgggaggatcactt
PPARA-B-R	AGCTaagtgatcctcccacctcggcctcccAAAGTGCtgggattccatgtgtgagccactgtgc
PPARA-C-F	CTAGtcccgggacactcagcagcgatggtgaGCATTTTggtttccttaaggcccagcaagactt
PPARA-C-R	AGCTaagtcttgctgggccttaaggaaaccAAAATGCtcaccatcgctgctgagtgtcccggga
PPARA-D-F	CTAGgcacagtcgctcatacttgtaatcccaGCACTTTgggaggccgaggcgggtggatcacaa
PPARA-D-R	AGCTttgtgatccacccgcctcggcctcccAAAGTGCtgggattacaagtatgagcgactgtgc
PPARA-E-F	CTAGggcgcatccctcctctcccacctcctgGCACTTCcagctgggtgtcccacatgttggatt
PPARA-E-R	AGCTaatccaacatgtgggacacccagctgGAAGTGCcaggaggtgggagaggagggatgcgcc
PPARA-F-F	CTAGcctgggcctctctgtgtttggttccaaGCACTTCccacctcaaactcccattttcaaacc
PPARA-F-R	AGCTggtttgaaaatgggagtttgaggtggGAAGTGCttggaaccaaacacagagaggcccagg
PPARA-G-F	CTAGtgctagccgctggtccccaggcacggtGCACTTTctccacctcctgcagcctccctgttg
PPARA-G-R	AGCTcaacagggaggctgcaggaggtggagAAAGTGCaccgtgcctggggaccagcggctagca
PPARA-H-F	CTAGtgctgggatttagatattttcaggcacCATTTTGacagcattcaggaaaacggttattga
PPARA-H-R	AGCTtcaataaccgttttcctgaatgctgtCAAAATGgtgcctgaaaatatctaaatcccagca
PTEN-A-F	CTAGtggccgctgtcactgcttgttgtttgcGCATTTTtttttaaagcatattggtgctagaaa

PTEN-A-R	AGCTtttctagcaccaatatgctttaaaaaAAAATGCgcaaacaacaagcagtgacagcggcca
RB1-A-F	CTAGaggaccctaacacagtatatcccaagtGCACTTTctaatgtttctgggtcctgaagaatt
RB1-A-R	AGCTaattcttcaggacccagaaacattagAAAGTGCacttgggatatactgtgttagggtcct
RB1-B-F	CTAGgtagcttcagctagcttttaggaaaatCACTTTGtctaactcagaattatttttaaaaag
RB1-B-R	AGCTctttttaaaaataattctgagttagaCAAAGTGattttcctaaaagctagctgaagctac
RB1CC1-A-F	CTAGgccataagtaatcagcaatcttcaaaaGCACTTTcagtggattggtcatctgggttctaa
RB1CC1-A-R	AGCTttagaacccagatgaccaatccactgAAAGTGCttttgaagattgctgattacttatggc
RB1CC1-B-F	CTAGagaggagtgttattgcatgctgataatCATTTTGagtttgcctcagtagatactaaagca
RB1CC1-B-R	AGCTtgctttagtatctactgaggcaaactCAAAATGattatcagcatgcaataacactcctct
RBBP7-A-F	CTAGttatggcttctttatcctctgattctaGCACTTTcaagtgagctgttgcgtactgtatca
RBBP7-A-R	AGCTtgatacagtacgcaacagctcacttgAAAGTGCtagaatcagaggataaagaagccataa
RBL1-A-F	CTAGgcctgatgtcacaacccaataaatgggGCACTTTcttcttttgtaaactattatcatttt
RBL1-A-R	AGCTaaaatgataatagtttacaaaagaagAAAGTGCcccatttattgggttgtgacatcaggc
RBL1-B-F	CTAGaatgttgttcttgtttctatgataaaaGCACTTTcagattgttctgcagaaagttggagc
RBL1-B-R	AGCTgctccaactttctgcagaacaatctgAAAGTGCttttatcatagaaacaagaacaacatt
RBL2-A-F	CTAGttacagcctgttagtaacatgaggggaCATTTTGgtgagaaatgggacttaactccttcc
RBL2-A-R	AGCTggaaggagttaagtcccatttctcacCAAAATGtcccctcatgttactaacaggctgtaa
RBL2-B-F	CTAGcccagattttgctgtatatttgtgataGCACTTTctacaatgtgaactttattaaataca
RBL2-B-R	AGCTtgtatttaataaagttcacattgtagAAAGTGCtatcacaaatatacagcaaaatctggg
RBL2-C-F	CTAGtgtctgagtgaacctgtataagtggagGCACTTTagggctgtaaaatgcatgattttgta
RBL2-C-R	AGCTtacaaaatcatgcattttacagccctAAAGTGCctccacttatacaggttcactcagaca
SMAD7-A-F	CTAGtataaacaagaaaaataaagaaaagatGCACTTTgctttaatataaatgcaaataacaaa
SMAD7-A-R	AGCTtttgttatttgcatttatattaaagcAAAGTGCatcttttctttattttcttgtttata
STAT3-A-F	CTAGaaataggaaggtttaaggagaatctaaGCATTTTagactttttttataaatagacttat
STAT3-A-R	AGCTataagtctatttataaaaaaaagtctAAAATGCttagattctccttaaaccttcctattt
STAT3-B-F	CTAGcacctatagctacatactcctggcattGCACTTTttaaccttgctgacatccaaatagaa
STAT3-B-R	AGCTttctatttggatgtcagcaaggttaaAAAGTGCaatgccaggagtatgtagctataggtg
STAT3-C-F	CTAGgcatggtggctcacgcctgtaatcccaGCACTTTgggaggccgaggcggatcataaggtc
STAT3-C-R	AGCTgaccttatgatccgcctcggcctcccAAAGTGCtgggattacaggcgtgagccaccatgc
STAT3-D-F	CTAGtacttctgctatctttgagcaatctggGCACTTTtaaaaatagagaaatgagtgaatgtg
STAT3-D-R	AGCTcacattcactcatttctctattttaAAAGTGCccagattgctcaaagatagcagaagta
TIMP2-A-F	CTAGtttccgtttggatttttgggggaggggAGCACTGtgtttatgctggaatatgaagtctga
TIMP2-A-R	AGCTtcagacttcatattccagcataaacaCAGTGCTcccctcccccaaaaatccaaacggaaa
TIMP2-B-F	CTAGttattacccttggtaggtattagacttGCACTTTtttaaaaaaaggtttctgcatcgtgg
TIMP2-B-R	AGCTccacgatgcagaaaccttttttaaaAAAGTGCaagtctaatacctaccaagggtaataa
TIMP2-C-F	CTAGctgggttagggcagggcctggaaatgtGCATTTTgcagaaacttttgagggtcgttgcaa
TIMP2-C-R	AGCTttgcaacgaccctcaaaagtttctgcAAAATGCacatttccaggccctgccctaacccag
TNXIP-A-F	CTAGtcttaaaagccatttttggagcctattGCACTGTgttctcctactgcaaatattttcata
TNXIP-A-R	AGCTtatgaaaatatttgcagtaggagaacACAGTGCaataggctccaaaaatggcttttaaga
TNXIP-B-F	CTAGggaattgattctaaggtgatgttcttaGCACTTTaattcctgtcaaatttttgttctcc
TNXIP-B-R	AGCTggagaacaaaaaatttgacaggaattAAAGTGCtaagaacatcaccttagaatcaattcc
TNXIP-C-F	CTAGgcacttggtcagtcactctcagccataGCACTTTgttcactgtcctgtgtcagagcactg
TNXIP-C-R	AGCTcagtgctctgacacaggacagtgaacAAAGTGCtatggctgagagtgactgaccaagtgc
TP53INP1-A-F	CTAGacctgttctttgtaattgggttgtggtGCATTTTgcactacctggagttacagttttcaa
TP53INP1-A-R	AGCTttgaaaactgtaactccaggtagtgcAAAATGCaccacaacccaattacaaagaacaggt
TP53INP1-B-F	CTAGagttttgtacacagactgaacaatacaGCACTTTgccaaaaatgagtgtagcattgttta
TP53INP1-B-R	AGCTtaaacaatgctacactcatttttggcAAAGTGCtgtattgttcagtctgtgtacaaaact
TP53INP1-C-F	CTAGattttttttctcaactaattgtgtactGCACTGTaaggtgaaaattagccatccattatt
TP53INP1-C-R	AGCTaataatggatggctaattttcaccttACAGTGCagtacacaattagttgagaaaaaaaat
TP53INP1-D-F	CTAGctattgtaaatattagtggtttacaatGCGCTTTagacatatttctttaaaatgcaagca

TP53INP1-D-R	AGCTtgcttgcattttaaagaaatatgtctAAAGCGCattgtaaaccactaatatttacaatag
TP53INP1-E-F	CTAGaagtattctctgaaacatggccaaaatGCATTTTatgagcttttttttttgctattgtaa
TP53INP1-E-R	AGCTttacaatagcaaaaaaaaaagctcatAAAATGCattttggccatgtttcagagaatactt
TP53INP1-F-F	CTAGtgaatctggtgatggggtggggccgtgGCACTTTctctgccacagctgttcttcacagtg
TP53INP1-F-R	AGCTcactgtgaagaacagctgtggcagagAAAGTGCcacggccccaccccatcaccagattca
TP53INP1-G-F	CTAGtaaatatattaggttatacattagtcaGCATTTTaaagacatttcttcccaagtacgaga
TP53INP1-G-R	AGCTtctcgtacttgggaagaaatgtctttAAAATGCtgactaatgtataacctaatatattta
TSG101-A-F	CTAGttttggtaaagactggcttttattaatGCACTTTctatcctctgtaaactttttgtgctg
TSG101-A-R	AGCTcagcacaaaaagtttacagaggatagAAAGTGCattaataaaagccagtctttaccaaaa
WEE1-A-F	CTAGgaattagacttgtatatcccactgggaGCACTTTgtaggcattgcatgaaccatgggatg
WEE1-A-R	AGCTcatcccatggttcatgcaatgcctacAAAGTGCtcccagtgggatatacaagtctaattc
